# Proconvertase Furin Is Downregulated in Postural Orthostatic Tachycardia Syndrome

**DOI:** 10.3389/fnins.2019.00301

**Published:** 2019-03-29

**Authors:** Jasmina Medic Spahic, Fabrizio Ricci, Nay Aung, Jonas Axelsson, Olle Melander, Richard Sutton, Viktor Hamrefors, Artur Fedorowski

**Affiliations:** ^1^Department of Clinical Sciences, Faculty of Medicine, Clinical Research Center, Lund University, Malmö, Sweden; ^2^Department of Internal Medicine, Skåne University Hospital, Malmö, Sweden; ^3^Institute for Advanced Biomedical Technologies, Department of Neuroscience, Imaging and Clinical Sciences, Università degli Studi “G. d’Annunzio” Chieti-Pescara, Chieti, Italy; ^4^The William Harvey Research Institute, NIHR Cardiovascular Biomedical Research Unit at Barts, Queen Mary University of London, London, United Kingdom; ^5^Department of Clinical Immunology and Transfusion Medicine, Karolinska University Hospital, Stockholm, Sweden; ^6^National Heart and Lung Institute, Imperial College London, London, United Kingdom; ^7^Department of Cardiology, Skåne University Hospital, Malmö, Sweden

**Keywords:** postural orthostatic tachycardia syndrome, inflammation, biomarkers, proteomics, proconvertase furin

## Abstract

**Background:** Postural Orthostatic Tachycardia Syndrome (POTS) is a cardiovascular autonomic disorder characterized by orthostatic intolerance and high prevalence among young women. The etiology of POTS is uncertain, though autoimmunity and inflammation may play an important role. We aimed to identify novel inflammatory biomarkers associated with POTS.

**Methods and Results:** In the Syncope Study of Unselected Population in Malmö (SYSTEMA) cohort, we identified 396 patients (age range, 15–50 years) with either POTS (*n* = 113) or normal haemodynamic response during passive head-up-tilt test (*n* = 283). Blood samples were analyzed using antibody-based Proximity Extension Assay technique simultaneously measuring 57 inflammatory protein biomarkers. The discovery algorithm was a sequential two-step process of biomarker signature identification by supervised, multivariate, principal component analysis and verification by univariate ANOVA with Bonferroni correction. POTS patients were younger (26 vs. 31 years; *p* < 0.001) and there was no significant difference in sex distribution (74% vs. 67% females, *p* = 0.24). PCA and Bonferroni-adjusted ANOVA identified proconvertase furin as the most robust biomarker signature for POTS. Plasma level of proconvertase furin was lower (6.38 vs. 6.58 of normalized protein expression units (NPX); *p* < 0.001 in POTS, compared with the reference group. Proconvertase furin met Bonferroni-adjusted significance criteria in both uni- and multivariable regression analyses.

**Conclusion:** Patients with POTS have lower plasma level of proconvertase furin compared with individuals with normal postural hemodynamic response. This finding suggests the presence of a specific autoimmune trait with disruption of immune peripheral tolerance in this hitherto unexplained condition. Further studies are needed for external validation of our results.

## Introduction

Postural orthostatic tachycardia syndrome (POTS) is a complex condition featuring signs of autonomic dysfunction with both cardiovascular and non-cardiovascular symptoms (Benarroch, 2012). Although typically multi-symptomatic, POTS is by definition characterized by an abnormally increased heart rate upon standing and symptoms of orthostatic intolerance without significant blood pressure decrease (Sheldon et al., 2015). The syndrome affects predominantly young women (70–80%) with increasing incidence in developed countries, but its etiology has not been established (Sheldon et al., 2015; Brinth et al., 2018). Aside from orthostatic intolerance, patient frequently report headache, palpitations, brain fog and fatigue, which is believed to be a consequence of both hyperadrenergic state and decreased blood flow to the brain (Benarroch, 2012; Li et al., 2014; Arnold et al., 2018).

As etiology of POTS is still unknown, effective treatment for this syndrome is yet to be developed. Therapeutic options available today have modest efficacy and focus only on alleviating symptoms, thus, further research is mandatory. It has been observed that some patients develop POTS after experiencing a febrile illness, presumably viral (Grubb, 2008; Li et al., 2014). This has led to the hypothesis of an autoimmune-mediated etiology of POTS, and recent case series of POTS following immune triggers like infection or vaccination (Blitshteyn, 2015; Brinth et al., 2015; Watari et al., 2018) support this hypothesis. It has already been established that activating autoantibodies (AAb) to the α1-adrenergic (α1AR), β1/2-adrenergic receptors (β1/2AR), and angiotensin-receptor type 1 can be found in serum from POTS patients but not in controls (Fedorowski et al., 2017; Yu et al., 2018). In current knowledge of the presence of autoantibodies and possible immunological triggers, exploration of expression of inflammatory mediators in POTS is required, both as a potential diagnostic tool and therapeutic target in this ill-understood condition for which there is no effective treatment.

In this study, we sought to discover inflammatory biomarkers associated with POTS in order to identify a signature, which could potentially be useful to understand the pathophysiology underlying the syndrome. We applied a new method of multiple-protein screening using oligonucleotide-labeled antibodies against selected serum proteins.

## Materials and Methods

### Study Population

The study was performed between September 2008 and May 2014 on a series of 545 consecutive patients aged 15–50 years enrolled in the ongoing Syncope Study of Unselected Population in Malmö (SYSTEMA) (Fedorowski et al., 2010). The age range was based on previous epidemiological studies on POTS incidence (Goodman, 2018). The recruited patients were referred to the tertiary syncope investigation unit at Skåne University Hospital in Malmö from primary and outpatient care clinics as well as hospitals in the southern region of Sweden due to unexplained syncope and/or symptoms of orthostatic intolerance. The referred patient underwent an initial diagnostic workup, typically including clinical history, resting, exercise and ambulatory prolonged electrocardiogram (Holter-ECG), transthoracic echocardiography, coronary and pulmonary angiography, brain imaging and encephalography, if requested by the referring physician. In the Syncope Unit, the patients were investigated by cardiovascular autonomic tests including head-up tilt testing (HUT), according to the European syncope guidelines available during this period (Moya et al., 2009). Blood samples were collected during the examination. The final study population included 113 POTS individuals and 283 controls with normal hemodynamic response during HUT as well as without prevalent cardiovascular disease (ischemic heart disease, heart failure, and stroke) or hypertension.

The study protocol conformed to the ethical guidelines of the 1975 Declaration of Helsinki and was approved by The Regional Ethical Review Board of Lund University (No 82/2008). All patients gave their written informed consent.

The PICO model was as follows: patients with unexplained syncope or orthostatic intolerance (Population), blood samples and HUT (Intervention), POTS patients versus controls (Comparison), and targeted protein biomarker discovery and haemodynamic response (Outcome).

### Examination Protocol

Patients discontinued cardiovascular drugs 48 h before the test, fasted for 2 h prior to examination and were allowed to drink water at will. The past medical history was explored using a standard study questionnaire. The patients were placed on a tilt table and a venous cannula was inserted in the forearm after which a rest period for at least 10 min was allowed before blood samples were collected through the cannula. As soon as the haemodynamic parameters were stable, a standard 70°HUT was carried out according to the Italian protocol recommended by ESC (Bartoletti et al., 2000; Moya et al., 2009). Beat-to-beat blood pressure and ECG were monitored continuously by a validated non-invasive photoplethysmographic method (Nexfin monitor; BMEYE, Amsterdam, Netherlands) with a wrist unit and finger cuff of appropriate size (Eeftinck Schattenkerk et al., 2009). POTS was defined as reproduction of symptoms of orthostatic intolerance (lightheadedness, dizziness, or discomfort) along with heart rate increase >30 beats/min or sinus tachycardia >120 beats/min during first 10 min of HUT; or increase >40 beats/min for those under 18 years of age, with a history of orthostatic intolerance for at least 6 months (Sheldon et al., 2015).

### Multiplex Protein Analysis

Plasma biomarkers were measured from supine blood samples (total volume: 30 ml) that had been first centrifuged, then stored as 16 × 250 μL aliquots of EDTA plasma in plastic thermotubes, and frozen at -80°C. For biomarker analysis, the samples were thawed and examined by the Proximity Extension Assay technique using the Olink Proteomics Proseek Multiplex Oncology I v1 96 × 96 reagents kit, which simultaneously measures 57 inflammatory and cancer-related human protein biomarkers in plasma (Supplementary Table S1). Briefly, a pair of oligonucleotide-labeled antibodies, Proseek probes, binds to the target protein in the plasma sample. When the two Proseek probes are in close proximity, a new polymerase-chain reaction (PCR) target sequence is formed by a proximity-dependent DNA polymerization event. This complex is subsequently detected and quantified using standard real-time PCR. The generated Normalised Protein Expression (NPX) unit is on a log2 scale, which means that a larger number represents a higher protein level in the sample. Additional information concerning limit of detection, reproducibility and validation is available at the Olink Proteomics website^1^.

### Statistical Analysis

For the statistical analyses, patients with available proteomics data and definitive diagnosis of POTS (*n* = 113) or normal hemodynamic response during HUT (*n* = 283), i.e., without orthostatic hypotension (Moya et al., 2009; Freeman et al., 2011) and abnormal postural tachycardia, as well as without prevalent cardiovascular disease (ischemic heart disease, heart failure, and stroke) or hypertension were selected. Missing data was imputed with multiple imputations by chained equations (MICE) approach to create 10 complete datasets. We used predictive mean matching for continuous variables, logistic regression for binary variables, and polytomous regression for categorical variables. All covariates were included in the imputation models. The maximum iteration was set at 20 and convergence was confirmed by visual examination of trace plots. The main characteristics of study population were calculated as mean and standard deviation for continuous variables and as percentages for categorical variables, both for the total study population, and separately for POTS-positive and -negative patients.

The discovery algorithm for the identification of potentially relevant biomarkers associated with the presence of POTS was a sequential two-step process of (i) biomarker signature identification by a supervised, multivariate, principal component analysis, and (ii) verification by univariate ANOVA with Bonferroni correction. This method has been previously validated in our hands (Johansson et al., 2018a,b).

After defining a minimal call rate <75%, we screened the biomarker panel through supervised principal component analysis, according to the algorithm first described by Hastie et al. (2013), which includes the following steps:

(1) For each biomarker, compute the standardized univariate logistic regression coefficient which represents the effect size for the outcome (presence or absence of POTS).
(2) Using an arbitrary effect size threshold θ from the list 0 ≤ θ_1_ < θ_2_ < 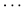 < θ_K_.
  (a) Form a reduced data matrix consisting of only those biomarkers whose univariate coefficient exceeds θ in absolute value, and compute the principal components of this matrix.
  (b) Use these principal components in a multivariate logistic regression model to predict POTS status.
(3) Select the threshold θ which gives the best predictive accuracy by 10-fold cross-validation.

Thereafter, for the verification of the selected biomarkers we applied a conservative univariate ANOVA approach, using a Bonferroni-adjusted significance level of *p* < 0.05/number of PCA-selected biomarkers. Box plots were generated to display the distribution of biomarker levels between groups.

Furthermore, we performed both univariate and multivariate ordinary least square linear regression and logistic regression models for bivariate correlation between plasma level of selected biomarkers and maximum orthostatic heart rate change (ΔHR) or POTS status, respectively. In multivariate regression models we adjusted for age and sex. Finally, we performed a quantile-regression analysis in order to identify differing relationships at different quartiles of HR changes during HUT. The mean estimates and standard errors of the beta-coefficients for the imputed datasets were combined with Rubin’s rules (Rubin, 1987). Statistical analyses were carried out using IBM SPSS Statistics version 25 (SPSS Inc., Chicago, IL, United States) and R Statistical Software (version 3.4.4; R Foundation for Statistical Computing, Vienna, Austria).

## Results

### Biomarker Signature Discovery

The dataset consisted of 396 patients (113 POTS and 283 controls). Baseline characteristics of the study population by POTS status are shown in Table 1. There was no significant difference in characteristics between the whole cohort and complete cases without missing data (Supplementary Table S2). Since the principal component analysis requires pairwise complete data, we did not include markers with high missingness, i.e., above 35%. This filter resulted in removal of 9 biomarkers: erythropoietin, interleukin-2, interferon-gamma, tumor necrosis factor, carcinoembryonic antigen, vascular endothelial statin, lipopolysaccharide-induced tumor necrosis factor (TNF)-alpha factor, myeloid differentiation primary response protein, MHC class I polypeptide-related sequence A. Univariate logistic regression was performed for each of the 48 biomarkers. Heatmap representation of the data showing the hierarchical clustering of the 48 biomarkers by POTS status is shown in Supplementary Figure S2.

**Table 1:** Baseline characteristics of the study population.

Characteristic	POTS- (*n* = 283)	POTS+ (*n* = 113)	*P*-value
Age	31.47 (9.85)	26.27 (8.41)	<0.001
Female sex, *n* (%)	189 (66.8)	83 (73.5)	0.241
BMI, Kg/m^2^	24.33 (4.14)	22.69 (3.50)	<0.001
SBP supine, mmHg	120.07 (14.16)	120.41 (14.20)	0.833
DBP supine, mmHg	69.98 (8.21)	70.22 (8.22)	0.788
HR supine, bpm	68.86 (11.87)	71.13 (11.57)	0.084
SBP HUT min, mmHg	112.34 (13.35)	107.58 (16.24)	0.003
DBP HUT min, mmHg	71.83 (9.11)	72.46 (10.58)	0.552
HR HUT max, bpm	84.77 (13.77)	112.41 (15.63)	<0.001
Smoking, *n* (%)	58 (20.5)	16 (14.2)	0.188


**P*-values for differences between the groups are shown as mean and SD for continuous variables and as percentages for categorical variables; DBP, diastolic blood pressure; HR, heart rate; HUT min/max, lowest/highest value during passive head-up tilt test; IHD, ischemic heart disease; POTS, postural orthostatic tachycardia syndrome; SBP, systolic blood pressure.*

The regression coefficients were then standardized by dividing the coefficient with its standard error. All possible thresholds (Standardized coefficient (θ) ranging from minimum to maximum with 0.05 increments) were used to select groups of biomarkers and construct principal components (PCs). The outcome variable (POTS status) was then regressed onto the 1st two PCs from each group of biomarkers using the binomial link function. This step identified the group of biomarkers which gave the best classification accuracy. The threshold that gave the best classification accuracy (POTS+ vs. controls) was selected by ten-fold cross-validation. The following four biomarkers reached this threshold: carbonic anhydrase IX; receptor tyrosine-protein kinase erbB-2; Fms-related tyrosine kinase 3 ligand; and proconvertase furin.

### Biomarker Verification

**FIGURE 1:**
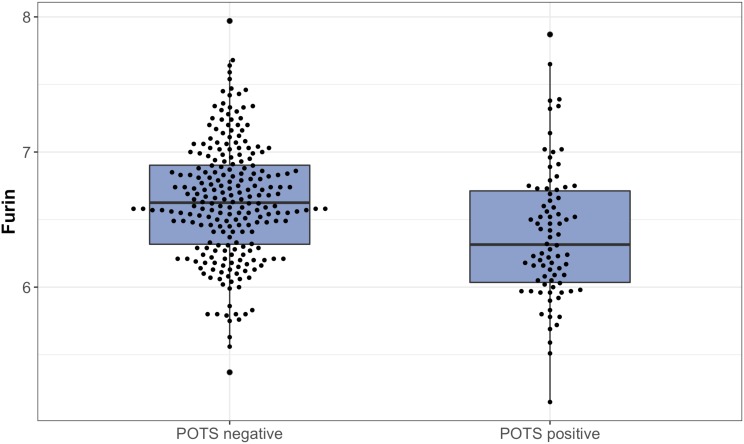
The plasma levels of proconvertase furin, expressed on a log2 scale, presented in relation to POTS status. Data are shown as a box and whisker plot with median in the box and the whiskers representing the 5th and 95th percentiles in relation to plasmatic biomarker levels.

All PCA-selected biomarkers except carbonic anhydrase IX differed significantly in pairwise comparison, but only proconvertase furin attained significance after Bonferroni correction (Figure 1 and Table 2). In multivariate regression analysis both POTS status and maximum orthostatic ΔHR were significantly associated with proconvertase furin (Tables 3, 4). Quantile regression analyses investigating the relationships between proconvertase furin and the quartiles of ΔHR did not reveal any obvious threshold effect or step function (Supplementary Figure S1). Finally, tertiles of proconvertase furin were inversely proportional to duration of symptoms (ANOVA, *p*-value for multiple comparisons = 0.021), defined as the time elapsed from the first symptom manifestation (presyncope or syncope) in patient’s history and the time of diagnostic examination (HUT).

**Table 2:** High throughput multiplex analysis biomarkers selected by supervised multivariate principal component analysis.

Biomarker	POTS+ (*n* = 113)	POTS- (*n* = 283)	*P*-value
FUR	6.38 (0.05)	6.58 (0.03)	0.000276ˆ*
CAIX	1.53 (0.09)	1.73 (0.06)	0.050109
Flt3L	7.76 (0.05)	7.89 (0.03)	0.012979
ErbB2HER2	7.79 (0.04)	7.92 (0.03)	0.026045


*Plasma concentrations of the assessed proteins are expressed on a log2-scale. Inter-group differences were assessed using analysis of variance method. ^∗^Bonferroni-corrected significant values (*p* < 0.012). CAIX, Carbonic anhydrase IX; ErbB2HER2, Receptor tyrosine-protein kinase erbB-2; Flt3L, Fms-related tyrosine kinase 3 ligand; FUR, furin.*

**Table 3:** Relationship between POTS status and selected biomarker in univariate and multivariate regression.

Biomarker	Univariate			Multivariate^∗^		
	*OR*	95% CI	*P*-value	*OR*	95% CI	*P*-value
FUR	0.81	0.73–0.92	<0.001	0.86	0.77–0.96	0.009


*^∗^Adjusted for age, sex.*

**Table 4:** Relationship between changes in heart rate during head-up tilt test and selected biomarker in univariate and multivariate regression.

Biomarker	Univariate			Multivariate^∗^		
	*β*	95% CI	*P*-value	*β*	95% CI	P-value
FUR	-7.0	-10.6 to -3.38	<0.001	-4.2	-7.9 to -0.5	0.03


*^∗^Adjusted for age, sex.*

## Discussion

In this study, we explored the inflammatory proteomic signature of POTS in order to elucidate pathophysiological mechanisms underlying this particular phenotype of cardiovascular autonomic dysfunction. We demonstrated that POTS is associated with lower circulating levels of proprotein convertases subtilisin/kexin type (PCSK)-3, i.e., proconvertase furin. Interestingly, the earlier the disease starts, the lower the proconvertase furin level.

Our findings confirm and further expand the growing body of evidence pointing to an immune dysregulation as the primary pathophysiological mechanism underlying POTS. However, it is noteworthy that pro-inflammatory cytokines and chemokines, i.e., interleukin-7, interleukin-12 and CXC motif chemokine 13, associated with systemic inflammatory diseases such as systemic lupus erythromatosus or rheumatoid arthritis were not increased among POTS-positive patients.

### The Role of Immune System in POTS

The presence of immunoglobulins activating and modulating multiple G-protein coupled receptors (GPCR) linked to the autonomic nervous system has been previously demonstrated in POTS patients (Fedorowski et al., 2017; Yu et al., 2018), as well as their ability to alter dose-response to the natural ligands *in vitro*. Patients suffering from POTS are most frequently young females of childbearing age (Li et al., 2014). Its onset is occasionally preceded by or associated with a viral-like illness or post vaccination which leads to the suspicion that autoimmunity may have an important role in these patients (Li et al., 2014; Brinth et al., 2015). Studies performed recently indicate that patients with POTS have higher prevalence of autoimmune markers and co-morbid autoimmune disorders compared with the general population (Blitshteyn, 2015). Accordingly, nearly one fourth of patients with POTS have positive ANA and one in three have some type of autoimmune markers (Blitshteyn, 2015). Moreover, a high proportion of POTS patients are seropositive for circulating antiganglionic acetylcholine receptor antibodies (Watari et al., 2018).

Finally, presence of antibodies against adrenergic and cholinergic receptors were confirmed in patients suffering from POTS in a number of studies, which could explain the adrenergic-related symptoms including exaggerated heart rate during orthostatic challenge (Ruzieh et al., 2017).

### Proconvertase Furin

Proconvertase furin is one of seven proprotein convertase family members that promote proteolytic maturation of proproteins (Barr et al., 1991). Ubiquitously expressed it has been reported to process a variety of secreted factors including cytokines and chemokines such as anti-inflammatory transforming growth factor (TGF)-β1 and secreted TNF-family receptors (Loetscher et al., 1990). Interestingly, previous evidence has suggested that proconvertase furin inhibition may result in a breakdown of peripheral tolerance (Pesu et al., 2008) and development of systemic autoimmune disease (Lisi et al., 2010). In experimental models of rheumatoid arthritis, exogenous proconvertase furin has been successfully used to harness autoimmunity (Lin et al., 2012). This is consistent also with recent findings reporting the association between high proconvertase furin levels and lower systemic activity disease in primary Sjögren’s syndrome (Ranta et al., 2018). Considering that proconvertase furin appears to be involved in maintaining immune homeostasis, it is important to note that only one POTS patient in our series had overt autoimmune comorbidity. Consequently, we may reasonably exclude that we have tracked down the effect of parallel autoimmune disorders on proconvertase furin in POTS population.

While the exact source of circulating levels of this protein in POTS patients remains unclear, we speculate that they may reflect a so far undetected viral activity. Indeed, cleavage of the human papilloma virus capsid protein L2 by proconvertase furin is necessary for infection (Richards et al., 2006), while the HIV-1 protein *Nef* is known to bind furin in order to sequester human leukocyte antigen-family receptors in the *trans*-Golgi network (Piguet et al., 2000). Presumably as a countermeasure, proconvertase furin is down-regulated during inflammation in a suppressor of cytokine signaling (Sox)-dependent manner (Guimont et al., 2007).

Taken together, further studies are warranted to confirm these findings in independent populations before proconvertase furin can be considered as an objective serum marker of POTS.

### Limitations

There is a number of important limitations that should be addressed. Firstly, inflammatory biomarkers have been sampled only at the time of POTS diagnosis; the lack of serial measurements prevent us to understand whether the alterations seen are a cause or a consequence of POTS, and whether there is any temporal correlation between proconvertase furin levels and the progression of the disease.

Secondly, this is a hypothesis-generating study aimed to discover alternative pathophysiological pathways involved in POTS, requiring external validation in another cohort.

Thirdly, data on important pro-inflammatory cytokines like IL-2, TNF-a, IFN is lacking.

Finally, in order to rule-out possible false positive signals, our findings should be verified and validated with alternative technologies enabling as much sensitive and robust detection and quantification of biomarkers; However, the use of a proximity assay with the requirement for a dual binding event which ensures minimal background signal, and the robust discovery algorithm, based on a sequential two-step process including principal component analysis and a very strict Bonferroni correction, would make a false positive result less likely.

## Conclusion

Proteomic profiling by proximity extension technique revealed an inflammation-specific biomarker fingerprint in POTS patients. Circulating levels of proconvertase furin are downregulated in POTS suggesting a complex and intriguing interplay between autoimmune activity and cardiovascular autonomic dysfunction.

## Ethics Statement

This study was carried out in accordance with the recommendations of local ethics guidelines, the Local Ethic Committee of Lund University, with written informed consent from all subjects. All subjects gave written informed consent in accordance with the Declaration of Helsinki. The protocol was approved by The Regional Ethical Review Board of Lund University (No. 82/2008).

## Author Contributions

FR, AF, JS, and NA had full access to all the data in the study and took responsibility of the data and accuracy of the data analysis. OM, AF, JS, VH, and JA contributed to the study conception and design. OM and AF contributed to the acquisition of data. All authors analyzed and interpreted the data. AF was the study supervisor. NA and FR did the statistical analysis. JS, FR, AF, and VH drafted the manuscript with critical revision for important intellectual content from all authors.

## Conflict of Interest Statement

The authors declare that the research was conducted in the absence of any commercial or financial relationships that could be construed as a potential conflict of interest.

## Footnotes

1 http://www.olink.com/products/document-download-center

## Supplementary Material

FIGURE S1 Heatmap visualization of the proteomics data showing the hierarchical clustering of 48 biomarkers by POTS status.

FIGURE S2 Quantile regression analysis. Furin regressed on 25th, 50th and 75th quantiles of delta HR max. The x axis is the quantile of delta HR max (black dots in the plots represent the regression coefficient at 0.25, 0.5 (median) and 0.75). The gray bands are the 95% CI of the quantile regression coefficient. The horizontal red and the two horizontal dotted lines are the ordinary least square (OLS) linear regression lines. What you can see here is that 95% CI of the coefficients from quantile regression overlaps widely with OLS lines indicating that Furin does not have differing effects on different quantiles of delta HR max.

TABLE S1 Immuno-oncology panel: biomarker list.

TABLE S2 Comparison of baseline characteristics between the whole cohort and complete cases without missing data.
