# Supplementary figures and images for: Proconvertase Furin Is Downregulated in Postural Orthostatic Tachycardia Syndrome
Source: Front Neurosci. 2019 Mar 29;13:301. doi: 10.3389/fnins.2019.00301 (PMC6455076; doi:10.3389/fnins.2019.00301)

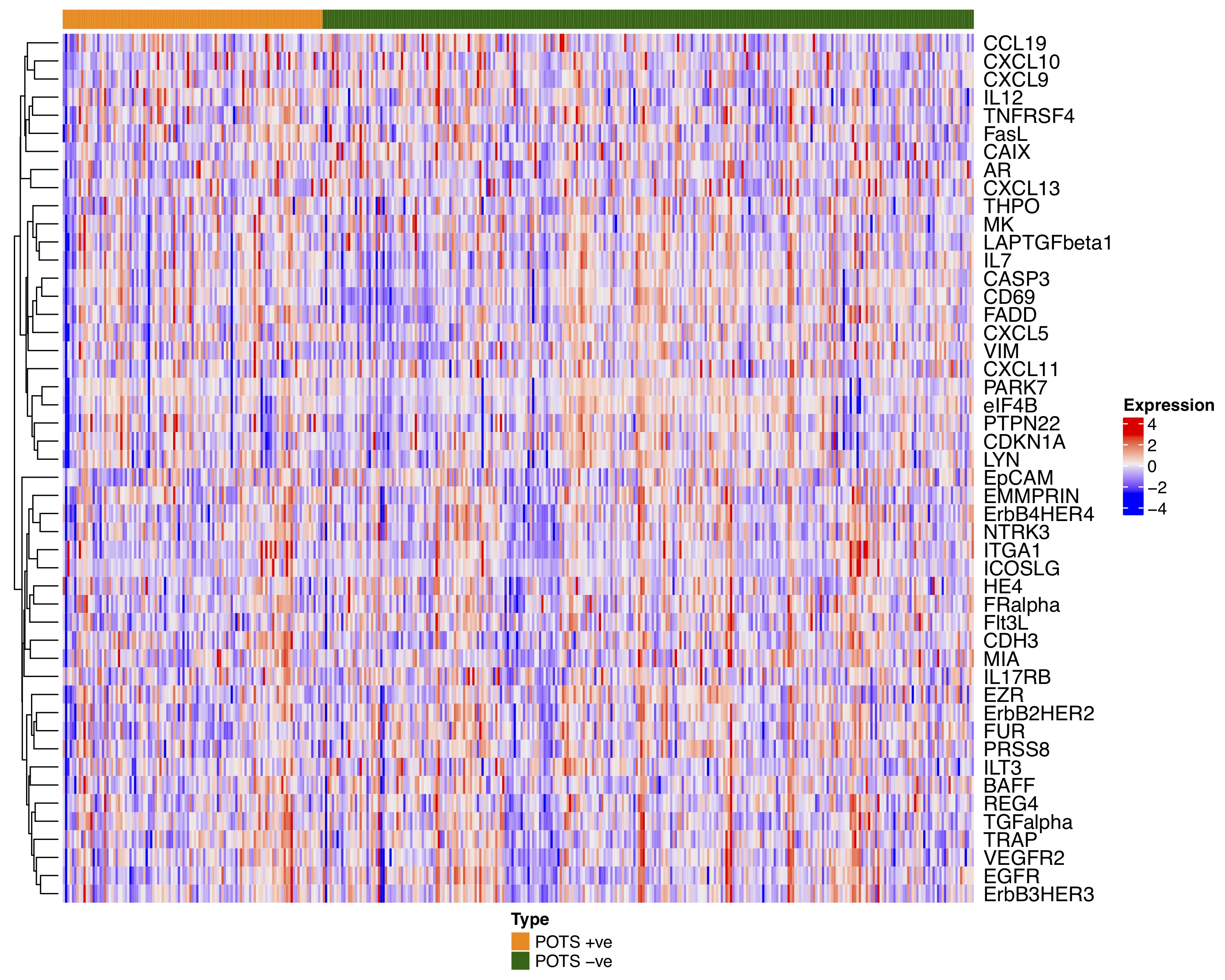

Supplement: FIGURE S1 — Heatmap visualization of the proteomics data showing the hierarchical clustering of 48 biomarkers by POTS status. [file Image_1.JPEG]

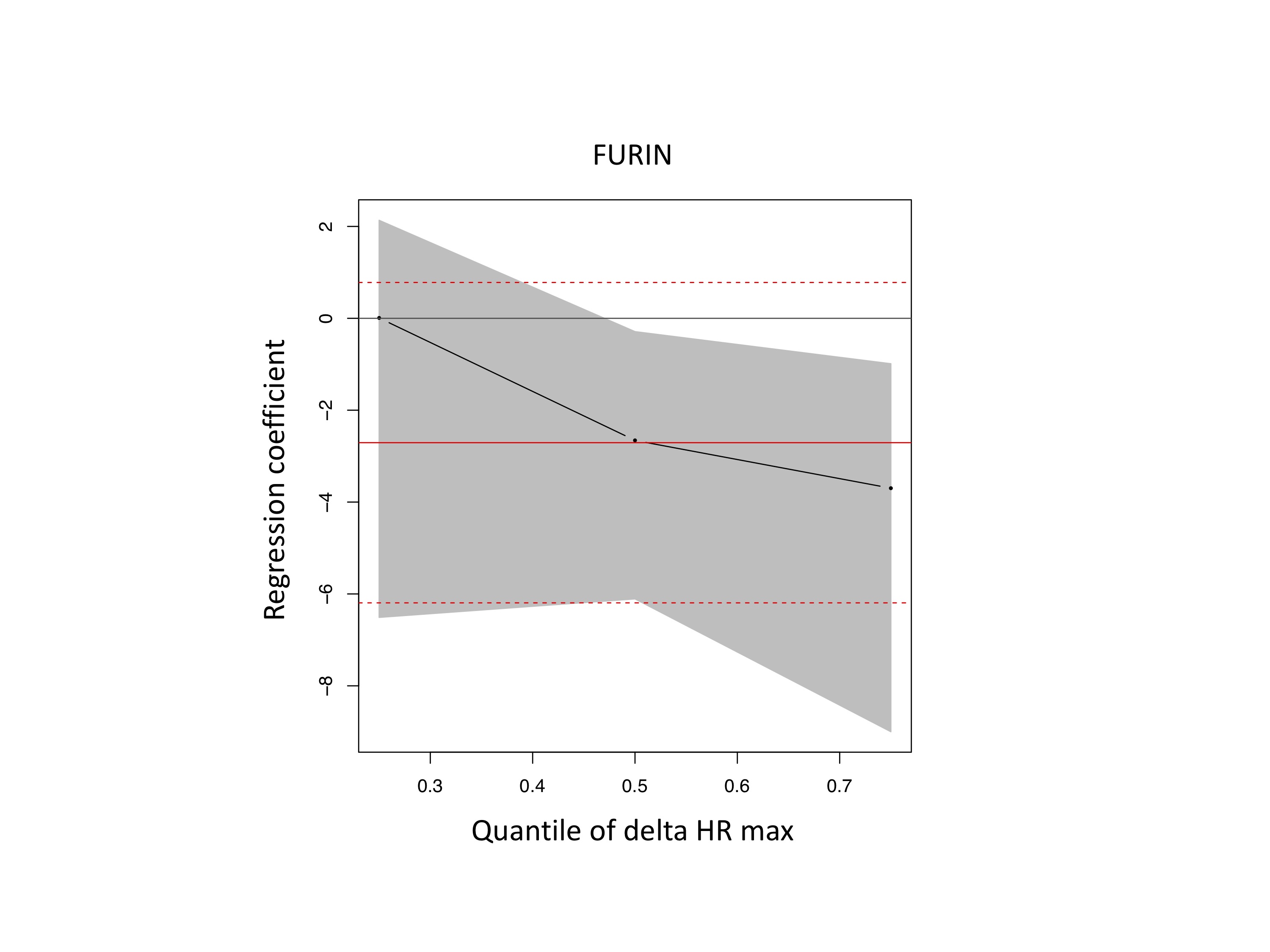

Supplement: FIGURE S2 — Quantile regression analysis. Furin regressed on 25th, 50th and 75th quantiles of delta HR max. The x axis is the quantile of delta HR max (black dots in the plots represent the regression coefficient at 0.25, 0.5 (median) and 0.75). The gray bands are the 95% CI of the quantile regression coefficient. The horizontal red and the two horizontal dotted lines are the ordinary least square (OLS) linear regression lines. What you can see here is that 95% CI of the coefficients from quantile regression overlaps widely with OLS lines indicating that Furin does not have differing effects on different quantiles of delta HR max. [file Image_2.JPEG]
