# Supplementary material for: Proconvertase Furin Is Downregulated in Postural Orthostatic Tachycardia Syndrome
Source: Front Neurosci. 2019 Mar 29;13:301. doi: 10.3389/fnins.2019.00301 (PMC6455076; doi:10.3389/fnins.2019.00301)
Supplement: TABLE S1 — Immuno-oncology panel: biomarker list. [file Table_1.DOCX]

**Table S1. Immuno-oncology panel: biomarker list**

| Amphiregulin (AR) |
| --- |
| B-cell activating factor (BAFF) |
| Cadherin-3 (CDH3) |
| Carbonic anhydrase IX (CAIX) |
| Carcinoembryonic antigen (CEA) |
| Caspase-3 (CAPS-3) |
| C-C motif chemokine 19 (CCL19) |
| C-X-C motif chemokine 10 (CXCL10) |
| C-X-C motif chemokine 11 (CXCL11) |
| C-X-C motif chemokine 13 (CXCL13) |
| C-X-C motif chemokine 5 (CXCL5) |
| C-X-C motif chemokine 9 (CXCL9) |
| Cyclin-dependant kinase inhibitor 1 (CDKN1A) |
| Early activation antigen CD69 (CD69) |
| Epidermal growth factor receptor (EGFR) |
| Epididymal secretory protein E4 (HE4) |
| Epithelial cell adhesion molecule (Ep-CAM) |
| Erythropoietin (EPO) |
| Eukaryotic translation initiation factor 4B (eIF-4B) |
| Extracellular matrix metalloproteinase inducer (EMMPRIN) |
| Ezrin (EZR) |
| Fas antigen ligand (FasL) |
| FAS-associated death domain protein (FADD) |
| Fms-related tyrosine kinase 3 ligand (Flt3L) |
| Folate receptor alpha (FR-alpha) |
| Furin (FUR) |
| ICOS ligand (ICOSLG) |
| Immunoglobulin-like transcript 3 (ILT-3) |
| Integrin alpha-1 (ITGA1) |
| Interferon gamma (IFN-gamma) |
| Interleukin-2 (IL-2) |
| Interleukin-12 (IL-12) |
| Interleukin-17 receptor B (IL-17RB) |
| Interleukin-7 (IL-7) |
| Latency-associated peptide transforming growth factor beta-1 (LAP TGF-beta 1) |
| Lipopolysaccharide-induced tumor necrosis factor-alpha factor (LITAF) |
| Melanoma-derived growth regulatory protein (MIA) |
| MHC class I polypeptide-related sequence A (MIC-A) |
| Midkine (MK) |
| Myeloid differentiation primary response protein MyD88 (MYD88) |
| NT-3 growth factor receptor (NTRK3) |
| Parkinson disease protein 7 (PARK7) |
| Prostasin (PRSS8) |
| Receptor tyrosine-protein kinase erbB-2 (ErbB2/HER2) |
| Receptor tyrosine-protein kinase erbB-3 (ErbB3/HER3) |
| Receptor tyrosine-protein kinase erbB-4 (ErbB4/HER4) |
| Regenerating islet-derived protein 4 (REG-4) |
| Tartrate-resistant acid phosphatase type 5 (TR-AP) |
| Thrombopoietin (THPO) |
| Transforming growth factor alpha (TGF-alpha) |
| Tumor necrosis factor (TNF) |
| Tumor necrosis factor receptor superfamily member 4 (TNFRSF4) |
| Tyrosine-protein kinase Lyn (LYN) |
| Tyrosine-protein phosphatase non-receptor type 22 (PTPN22) |
| Vascular endothelial growth factor receptor 2 (VEGFR-2) |
| Vascular endothelial statin (VE-statin) |
| Vimentin (VIM) |

|  | **POTS-** | | | **POTS+** | | |
| --- | --- | --- | --- | --- | --- | --- |
| **Characteristic** | **Complete cases** | **Entire cohort with imputed data** | **P-value** | **Complete cases** | **Entire cohort with imputed data** | **P-value** |
| n | 183 | 283 |  | 73 | 113 |  |
| Age (mean (sd)) | 31.88 (9.33) | 31.47 (9.85) | 0.654 | 26.53 (8.26) | 26.27 (8.41) | 0.836 |
| Female sex (%) | 118 (64.5) | 189 (66.8) | 0.680 | 53 (72.6) | 83 (73.5) | 1.000 |
| BMI (mean (sd)) | 24.40 (3.76) | 24.33 (4.14) | 0.849 | 22.78 (3.86) | 22.69 (3.50) | 0.876 |
| Systolic BP supine (mean (sd)) | 120.81 (12.96) | 120.07 (14.16) | 0.572 | 122.41 (14.32) | 120.41 (14.20) | 0.350 |
| Diastolic BP supine (mean (sd)) | 69.68 (7.41) | 69.98 (8.21) | 0.697 | 71.16 (8.08) | 70.22 (8.22) | 0.443 |
| Heart rate supine (mean (sd)) | 68.67 (12.23) | 68.86 (11.87) | 0.868 | 71.01 (11.70) | 71.13 (11.57) | 0.946 |
| Systolic BP HUT min (mean (sd)) | 111.89 (12.36) | 112.34 (13.35) | 0.712 | 108.52 (17.33) | 107.58 (16.24) | 0.709 |
| Diastolic BP HUT min (mean (sd)) | 71.09 (8.88) | 71.83 (9.11) | 0.391 | 73.63 (10.91) | 72.46 (10.58) | 0.468 |
| Hearte rate HUT min (mean (sd)) | 84.71 (13.88) | 84.77 (13.77) | 0.966 | 113.23 (16.29) | 112.41 (15.63) | 0.730 |
| Current smoking (%) | 44 (24.0) | 58 (20.5) | 0.429 | 8 (11.0) | 16 (14.2) | 0.680 |

**Table S2. Comparison of baseline characteristics between the whole cohort and complete cases without missing data.**
